# Functional Identification of *Proteus mirabilis eptC* Gene Encoding a Core Lipopolysaccharide Phosphoethanolamine Transferase

**DOI:** 10.3390/ijms15046689

**Published:** 2014-04-21

**Authors:** Eleonora Aquilini, Susana Merino, Yuriy A. Knirel, Miguel Regué, Juan M. Tomás

**Affiliations:** 1 Department Microbiology and Parasitology, Faculty Pharmacy, University of Barcelona, Av. Joan XXIII s/n, 08028 Barcelona, Spain; E-Mail: eaquilini@ub.edu; 2 Department Microbiology, Faculty Biology, University Barcelona, Diagonal 643, 08071 Barcelona, Spain; E-Mail: smerino@ub.edu; 3 N.D. Zelinsky Institute of Organic Chemistry, Russian Academy of Sciences, Moscow 119991, Russia; E-Mail: yknirel@gmail.com

**Keywords:** *Proteus mirabilis*, core lipopolysaccharide, phosphoethanolamine

## Abstract

By comparison of the *Proteus mirabilis* HI4320 genome with known lipopolysaccharide (LPS) phosphoethanolamine transferases, three putative candidates (PMI3040, PMI3576, and PMI3104) were identified. One of them, *eptC* (PMI3104) was able to modify the LPS of two defined non-polar core LPS mutants of *Klebsiella pneumoniae* that we use as surrogate substrates. Mass spectrometry and nuclear magnetic resonance showed that *eptC* directs the incorporation of phosphoethanolamine to the *O*-6 of l-*glycero*-d-*mano*-heptose II. The eptC gene is found in all the *P. mirabilis* strains analyzed in this study. Putative *eptC* homologues were found for only two additional genera of the *Enterobacteriaceae* family, *Photobacterium* and *Providencia*. The data obtained in this work supports the role of the eptC (PMI3104) product in the transfer of PEtN to the *O*-6 of l,d-HepII in *P. mirabilis* strains.

## 1. Introduction

Gram-negative motile and frequently swarming bacteria of the genus *Proteus* from the family *Enterobacteriaceae* are usually found in soil, water, and the intestines of human and animals. The genus includes five species (*P. mirabilis*, *P. penneri*, *P. vulgaris*, *P. myxofaciens*, and *P. hauseri*) and three genomospecies [1]. Among these species, *P. mirabilis*, *P. vulgaris*, and *P. penneri* are the most common opportunistic pathogens [2]. *P. mirabilis* is frequently associated with urinary tract infections (UTI) in individuals with functional or anatomical abnormalities of the urinary tract or long-term catheterized patients. Complications arising from *P. mirabilis* infections include bladder and kidney stone formation, catheter obstruction by encrusting biofilms, and bacteremia [3]. Identified virulence factors include swarming, fimbriae, urease, hemolysin, and iron acquisition systems. Signature-tagged mutagenesis [4] has allowed the additional identification of an extracellular metalloprotease, several putative DNA binding regulatory, cell-envelope related, and plasmid encoded proteins.

In the lipopolysaccharide (LPS) of *P. mirabilis*, as in other members of the family *Enterobacteriaceae*, three domains can be recognized: lipid A, an endotoxic glycolipid; an *O*-polysaccharide chain or *O*-antigen (*O*-PS); and a core oligosaccharide (OS) domain, linking lipid A and *O*-PS. The chemical structure of the *P. mirabilis* lipid A has been determined for one strain showing the presence of a residue of 4-amino-4-deoxy-l-arabinose (l-Ara4N) substituting the phosphate at position 1 of lipid A, this l-AraN modification has been related to the high intrinsic *P. mirabilis* resistance to polymyxin B and related cationic antimicrobial peptides.

Among *P. mirabilis* and *P. vulgaris*, 60 *O*-serogroups have been recognized and the gene clusters involved in the biosynthesis of *P. mirabilis* serogroups O3, O10, O23, O27, and O47 have been reported [5]. In most *P. mirabilis O*-serogroups studied so far, the *O*-PSs contain acidic or both acidic and basic components, such as uronic acids, and their amides with amino acids, including lysine, and phosphate groups (reviewed in [6]).

**Figure 1.**
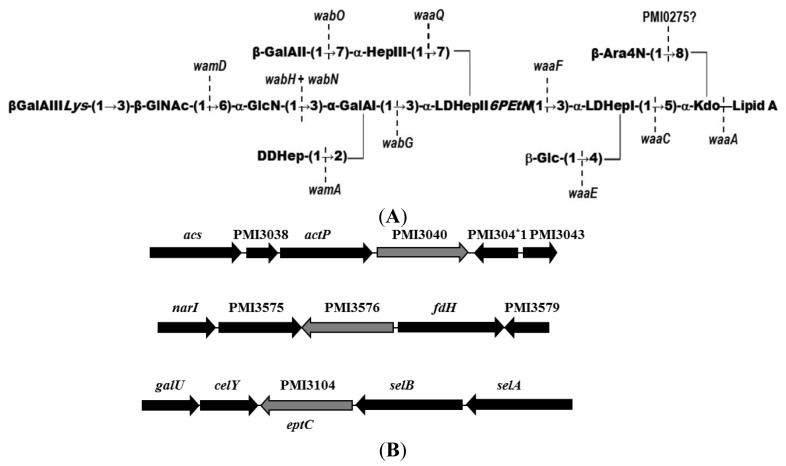
(**A**) *P. mirabilis* HI4320 core OS structure (50) and genes involved in core biosynthesis (2,3). 3-Deoxy-d-manno-oct-2-ulosonic acid (Kdo), l-glycero-d-manno-heptose (l,d-Hep), d-glycero-d-manno-heptose (D,D-Hep), glucosamine (GlcN) galactunonic acid (GalA), *N*-acetylglucosamine (GlcNAc), glucose (Glc), 4-amino-4-deoxy-l-arabinose (l-Ara4N), phosphoethanolamine (PEtN), and lysine (Lys); (**B**) Location in the genome of strain HI4320 of *eptC* (PMI3104) and putative PEtN transferases PMI3040 and PMI3576.

The core OS structure of the *P. mirabilis* genome strain HI4320 has been recently reported [7] (Figure 1). This structure up to the first outer-core galacturonic acid residue (d-GalA I) is shared by 11 *P. mirabilis* strains and also by several *P. vulgaris* and *P. penneri* serogroups. This common fragment is also found in the core LPS of *Klebsiella pneumoniae* and *Serratia marcescens*, but without the Ara4N and phosphoethanolamine (PEtN) residues [8,9] (Figure 1). Some *P. mirabilis* core-OS structures contain unusual residues such as quinovosamine, an open-chain form of *N*-acetyl-galatosamine, or amide linked amino acids. Recently, most of the genes involved in the biosynthesis of the sugar backbone of the core LPS from several *P. mirabilis* strains have been identified and characterized [10,11]. Little is known about the role of the non-sugar charged residues or groups in the core OS. In this study we identify a gene, *eptC*, essential for core LPS modification with PEtN.

## 2. Results and Discussion

### 2.1. Identification of Putative LPS PEtN Transferases

**Figure 2.**
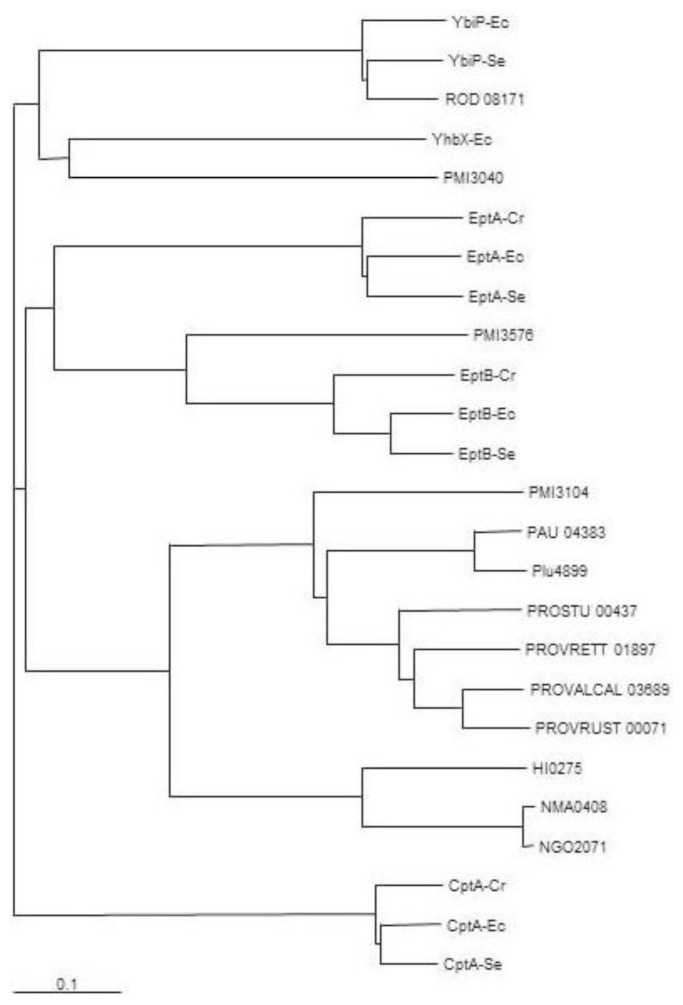
Phylogenetic tree of selected known and putative PEtN transferases. Shown are proteins from *P. mirabilis* HI4320 (PMI3040, PMI3576, PMI3104 [*eptC*]), *E. coli* MG1655 (YbiP-Ec, YhbX-Ec, CptA-Ec, EptA-Ec, EptB-Ec), *S. enterica* LT2 (YbiP-Se, CptA-Se, EptA-Se, EptB-Se), *Citrobacter rodentium* ICC168 (ROD 08171, CptA-Cr, EptA-Cr, EptB-Cr), *Photorhabdus asymbiotica asymbiotica* ATCC 43949 (PAU 04383), *Pho. luminescnes laumondii* TT01 (Plu4899), *Providencia stuartii* ATCC 25827 (PROSTU 00437), *Providencia rettgeri* DSM 1131 (PROVRETT 01897), *Providencia alcalifaciens* DSM 30120 (PROVALCAL 03689), *Providencia rustigianii* DSM 4541 (PROVRUST 00071), *Haemophilus influenzae* Rd KW20 (HI0275), *Neisseria meningitidis* Z2491 (NMA0408), and *N. gonorrhoeae* FA 1090 (NGO2071). Note: The separation and the size of the lanes are relative to the similarity degree according to the program used (the scale bar indicates an evolutionary distance of 0.1 amino acid substitutions per position).

Previous work done in *S. enterica* LT2 allowed the identification of several LPS PEtN transferases by similarity to *lpt-3* gene encoded protein Lpt3 (NMB2010) of *Neisseria meningitidis* [12]. This protein of *N. meningitdis* is responsible for the transfer of a PEtN residue to the *O*-2 of l,d-Hep II [12]. A similar search was performed for *P. mirabilis* genome strain HI4320 leading to the identification of PMI3040 (*e* value 1 × 10^−19^, 28% identity, 48% similarity) and PMI3576 (*e* value 6 × 10^−19^, 25% identity, 42% similarity). PMI3040 and PMI3576 shared with other known PEtN transferases the presence of a sulfatase domain. PMI3040 showed significant levels of amino acid identity and similarity to *E. coli* MG1665 YhbX (*e* value 1 × 10^−84^, 33% identity, 52% similarity, 94% coverage), YbiP (*e* value 3 × 10^−37^, 30% identity, 48% similarity, 57% coverage) and CptA (*e* value 6 × 10^−27^, 23% identity, 43% similarity, 89% coverage) (Figure 2).

**Figure 3.**
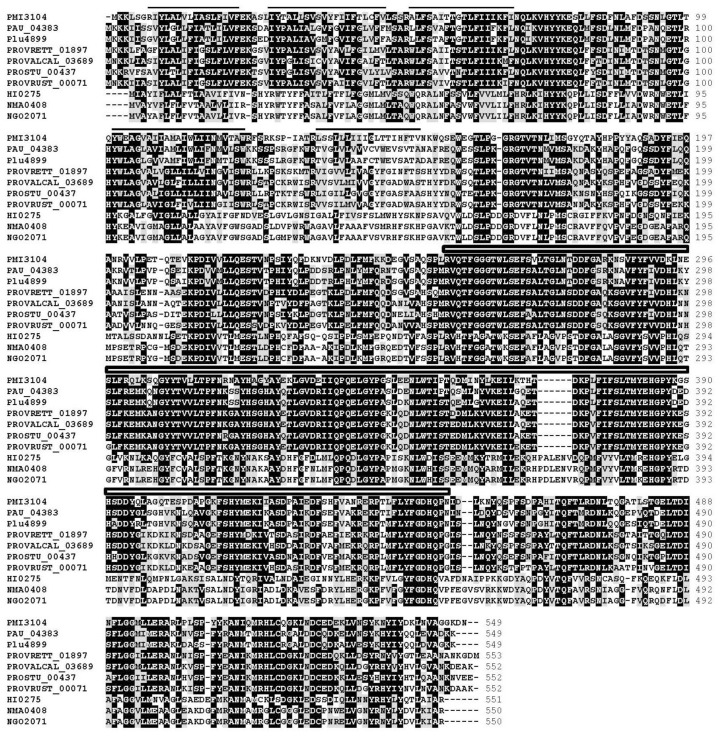
Amino acid alignment among known and putative proteins responsible for the transfer of PEtN to the *O*-6 of l,d-HepII. *P. mirabilis* PMI3104 (EptC), *Photorhabdus asymbiotica asymbiotica* ATCC 43949 (PAU_04383), *Pho. luminescnes laumondii* TT01 (Plu4899), *Providencia rettgeri* DSM 1131 (PROVRETT_01897), *Prov. alcalifaciens* DSM 30120 (PROVALCAL_03689), *Prov. stuartii* ATCC 25827 (PROSTU_00437), *Prov. rustigianii* DSM 4541 (PROVRUST_00071), *Haemophilus influenzae* Rd KW20 (HI0275), *Neisseria meningitidis* Z2491 (NMA0408), and *N. gonorrhoeae* FA 1090 (NGO2071). Black lines denote putative transmembrane regions and the box denotes amino acid residues similar to the sulfatase domain in PMI3104 (EptC). Numbers indicated the GenBank protein sequences; Identical amino acids are shown on a black background; and similar residues are shown on a gray background.

Similar results were found with *S. enterica* Typhimurium LT2 CptA (STM4118) and YbiP (STM08354) (Figure 2). While no function has been established for YhbX and YbiP, CptA is a PEtN transferase responsible for the linkage of PEtN to phosphorylated l,d-Hep I residue of the inner core in *S. enterica* [13]. PMI3576 shared significant levels of amino acid identity and similarity to EptB proteins from *E. coli* MG1665 (*e* value 0, 51% identity, 70% similarity, 98% coverage) and *S. enterica* LT2 (*e* value 0, 50% identity, 71% similarity, 96% coverage), and *Citrobacter rodentium* ICC168. PMI3576 also showed similarity to EptA proteins from *E. coli* and *S. enterica* with *e* values of 2 × 10^−43^ and 6 × 10^−47^, respectively (Figure 2). In *E. coli* EptB has been shown to transfer a PEtN moiety to the 3-deoxy-d-manno-oct-2-ulosonic II (Kdo II) residue of inner core LPS [14], and in *S. enterica* EptA, also known as PmrC, transfers PEtN to the phosphate at the 1 and/or 4′ positions of lipid A [15]. In *Neisseria meningitidis*, *N. gonorrhoeae*, and *Haemophilus influenza*, another PEtN residue is found substituting the *O*-6 position of l,d-Hep II [12]. A highly conserved Lpt6 protein, *N. meningitides* (NMA0408), *N. gonorrhoeae* (NGO2071), and *H. influenza* (HI0275), is required for the transfer of this PEtN. The whole genome of *P. mirabilis* HI4320 was analyzed by BLAST search for putative proteins being similar to NMA0408 allowing the identification of PMI3104 (*e* value 2 × 10^−99^, 33% identity, 53% similarity). Similar levels of similarity were found for the NMA0408 homologues NGO2071 and HI0275 (Figure 2). The PMI3104 shared with these proteins the presence of several predicted transmembrane regions before a sulfatase domain (Figure 3).

PMI3104 showed high levels of identity (75% to 77%) along the entire protein with putative homologues from *Photorhabdus asymbiotica*, *Pho. luminescens*, *Providencia rettgeri*, *Prov. alcalifaciens*, and *Prov. rustigianii* (Figures 2 and 3), but not to other members of the *Enterobacteriaceae* family. The three genera containing PMI3104 or its homologues are closely related at the phylogenetic level.

### 2.2. Identification of a Core LPS PEtN Transferase

Since the core LPS isolated from *P. mirabilis* strains present a moiety of PEtN substituting the *O*-6 position of l,d-Hep II (HepII6-PEtN) [6], it was hypothesized that PMI3104 would be the enzyme involved in this PEtN transfer and hence named *eptC*, for ethanolamine phosphate transferase C. On the basis of carbohydrate backbone composition and structure identity between the core LPS of *K. pneumoniae* and *P. mirabilis* up to the first residue of the outer core [9,10] (Figure 1), we decided to use the LPS from non-polar mutants *K. pneumoniae* 52145Δ*wabH* and *K. pneumoniae* 52145Δ*wabG* [9,16] as surrogate LPS acceptors to test *eptC* activity. This new approach has the advantage that we look for a positive trait as the addition to the mutant LPS of a particular residue, instead of the mutagenesis studies to determine the function of core LPS biosynthetic genes usually employed. This is only possible, as in our case, if a proper surrogate LPS acceptor is available.

**Figure 4.**
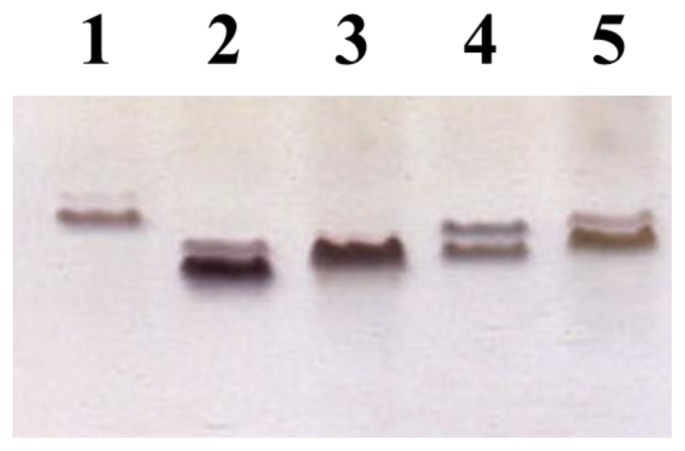
LPS electrophoretic pattern in Tricine-SDS-PAGE. LPS samples isolated from *K. pneumoniae* 52145 wild type (Lane 1), *K. pneumoniae* 52145Δ*wabG* (Lane 2), *K. pneumoniae* 52145Δ*wabG* plus pBAD18-Cm-*eptC* (Lane 3), *K. pneumoniae* 52145Δ*wabH* (Lane 4), and *K. pneumoniae* 52145Δ*wabH* plus pBAD18-Cm-*eptC* (Lane 5). Samples were obtained from strains grown under inducing conditions.

To guarantee the inducible expression of the *eptC* gene, plasmid pBAD18-Cm-*eptC* was constructed and electroporated into the two *K. pneumoniae* mutants 52145Δ*wabH* [9] and *K. pneumoniae* 52145Δ*wabG* [16]. LPS from *K. pneumoniae* 52145Δ*wabG* (+pBAD18-Cm-*eptC* grown under arabinose promoter inducing conditions) migration was slower than that of LPS from *K. pneumoniae* 52145Δ*wabG* grown under the same conditions (Figure 4, lanes 2 and 3). Similar results were obtained when comparing LPS from *K. pneumoniae* 52145Δ*wabH* (+pBAD18-Cm-*eptC*) and *K. pneumoniae* 52145Δ*wabH* always grown under arabinose promoter inducing conditions (Figure 4, lanes 4 and 5). No changes were observed in the LPS migration when the cells were grown under non induced conditions.

**Figure 5.**
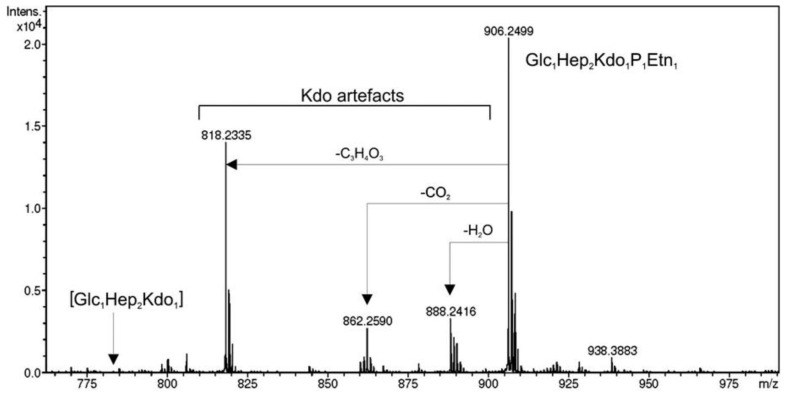
High-resolution electrospray ionization mass spectrum of core OS obtained by mild acid hydrolysis from LPS isolated from *K. pneumoniae* 52145Δ*wabG* harboring plasmid pBAD18-Cm-*eptC*.

The spectrometry (ESI-MS) spectrum of *K. pneumoniae* 52145Δ*wabG* core oligosaccharide showed a pseudo-molecular ion (M–H)– at *m*/*z* 783.37 which was in agreement with the calculated average molecular weight (783.67) of the expected molecular structure, with one hexose, two heptoses and one Kdo unit as previously reported [16]. The identical result was obtained when the core oligosaccharide of *K. pneumoniae* 52145Δ*wabG* (+pBAD18-Cm-*eptC*) was obtained from cells grown under non-induced conditions. To unambiguously test the *eptC* requirement for PEtN transfer, LPS from cells of 52145Δ*wabG* (+pBAD18-Cm-*eptC*) grown under arabinose promoter inducing conditions was isolated from dried cells by phenol-water extraction and purified (see Experimental Section). Mild acid degradation of this LPS resulted in a core fraction purified by gel-permeation chromatography on Sephadex G-50. High-resolution negative ion electrospray ionization mass spectrum of this fraction showed a major [M–H]^−^ ion peak at *m*/*z* 906.2499 for a compound corresponding to an OS composed of two heptose residues and one residue each of hexose, Kdo and PEtN (Hex_1_Hep_2_Kdo_1_P_1_EtN_1_) (calculated ion mass 906.2497 amu). In addition, a number of lower-molecular mass ions were observed in the spectrum at *m*/*z* 888.2416, 862.2590, and 818.2335 which were assigned to various Kdo artifacts generated by loss of H_2_O, CO_2_, and C_3_H_4_O_3_, respectively (Figure 5). No non-phosphorylated OS was detected.

**Figure 6.**
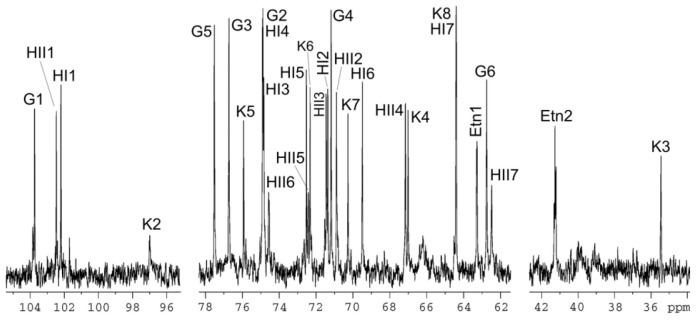
^13^C-NMR of core OS obtained by mild acid hydrolysis from LPS isolated from *K. pneumoniae* 52145Δ*wabG* harboring plasmid pBAD18-Cm-*eptC*.

The ^13^C-NMR spectrum of the OS (Figure 6) showed signals for four anomeric carbons at δ 97.0–103.7, one C–CH_2_–C group (*C*-3 of Kdo) at δ 35.4, four HOCH_2_–C groups (*C*-6 of Hex, *C*-7 of Hep, *C*-8 of Kdo) at δ 62.5–64.4, other sugar carbons in the region of δ 67.0–77.5, and one PEtN group at δ 41.2 (CH_2_N) and 63.3 (CH_2_O). The ^1^H-NMR spectrum contained, *inter alia*, signals for three anomeric protons at δ 4.55–5.34 and a PEtN group at 3.31 (CH_2_N). The ^31^P-NMR spectrum showed one signal for a monophosphate group at δ 0.5.

The ^1^H-NMR spectrum of the OS was assigned using two-dimensional COSY and TOCSY experiments, and then the ^13^C-NMR spectrum was assigned using a two-dimensional ^1^H-, ^13^C-HSQC experiment. The assigned ^1^H- and ^13^C-NMR chemical shifts (Table 1) were in full agreement with the carbohydrate backbone composition and structure expected for the core OS derived from *K. pneumoniae wabG* deletion mutant [16]. The signals for H-6 and C-6 of the terminal heptose residue (l,d-Hep II) were shifted downfield to δ 4.57 and 74.6, as compared with their positions in the non-substituted heptose at δ 4.04 and 70.4, respectively.

These data defined the site of phosphorylation at position *O*-6 of l,d-Hep II and, hence, the OS has the following structure:


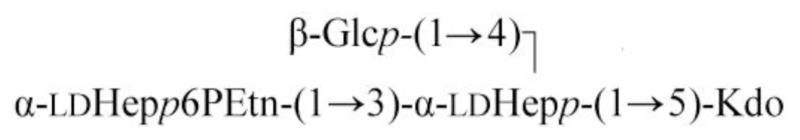


### 2.3. eptC Gene Distribution

**Figure 7.**
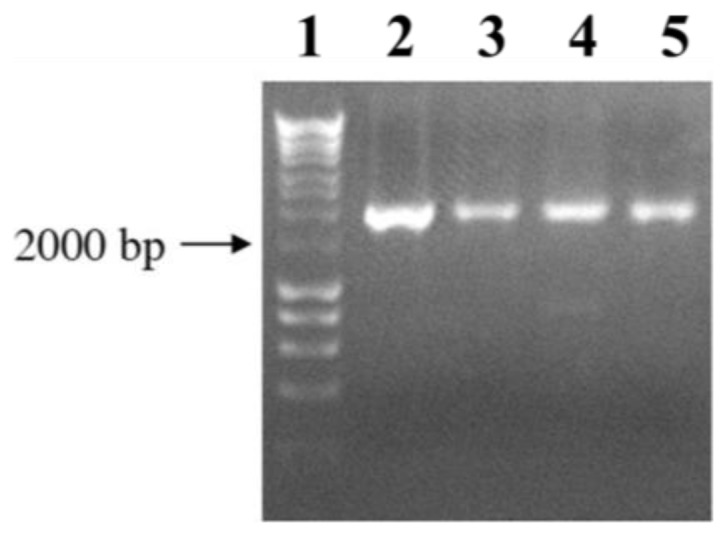
PCR amplification using oligonucleotides pair F1*eptC*-R1*eptC* and DNA template from *P. mirabilis* strains R110 (Lane 2), 50/57 (Lane 3), 51/57 (Lane 4), and TG83 (Lane 5). Lane 1, molecular weight standard.

Since the presence of a PEtN moiety linked to the *O*-6 of the l,d-Hep is a common feature of the core LPS of the studied *P. mirabilis* strains [17] the *eptC* gene should be present in these strains. To confirm the above hypothesis, a collection of *P. mirabilis* strains obtained from Z. Sydorckzyk (National Microbiology Institute, Warsaw, Poland) were used. Genomic DNA from these strains was used as template for amplification with oligonucleotides F1*eptC* and R1*eptC*. Fragments of about 2000 bp were amplified for all the strains studied, as exemplified by the results for the four strains shown in Figure 7.

Sequencing of the amplified fragments confirmed the presence of the *eptC* gene. In addition, in all the amplified fragments the presence of a sequence coding for the *C*-terminal amino acid residues of *celY* (PMI3103) gene, encoding a putative cellulase, were found adjacent to *eptC*, suggesting that in the analyzed strains the *eptC* location in their genome is the same as that found in genome strain HI4320 (Figure 1). In agreement with the EptC function as the transferase responsible for the linkage of a PEtN moiety to the *O*-6 position of l,d-Hep II, the *eptC* gene is found in all the *P. mirabilis* strains analyzed in this study. Within the *Enterobacteriaceae*, *eptC* homologues appear to be limited to species of the *Proteus* phylogenetic related genus *Photobacterium* and *Providencia* (Figure 2), and recently the presence of a PEtN moiety linked to the *O*-6 position of l,d-Hep II was shown in *Prov. alcalifaciens* O8 and O35, and *Prov. stuartii* O49 [18]. Further work will be necessary to understand the reasons for the presence of this common feature in these three closely related genera.

## 3. Experimental Section

### 3.1. Bacterial Strains, Plasmids, and Growth Conditions

The bacterial strains and plasmids used in this study are listed in Table 2. All strains were routinely grown in in LB medium (per liter, 0.5 g NaCl, 5.0 g yeast extract, 10.0 g tryptone) or on LB agar (15.0 g·L^−1^ agar). Ampicillin (100 μg·mL^−1^), chloramphenicol (20 μg·mL^−1^), and kanamycin (25 μg·mL^−1^) were added to the different media when required.

### 3.2. General DNA Methods

General DNA manipulations were done essentially as previously described [21]. DNA restriction endonucleases, T4 DNA ligase, *E. coli* DNA polymerase (Klenow fragment), and alkaline phosphatase were used as recommended by the Sigma-Aldrich (St. Louis, MO, USA).

### 3.3. DNA Sequencing and Computer Analysis of Sequence Data

Double-stranded DNA sequencing was performed by using the dideoxy-chain termination method [22] from PCR amplified DNA fragments with the ABI Prism dye terminator cycle sequencing kit (PerkinElmer, Barcelona, Spain). Oligonucleotides used for genomic DNA amplifications and DNA sequencing were purchased from Sigma-Aldrich (St. Louis, MO, USA). Deduced amino acid sequences were compared with those of DNA translated in all six frames from nonredundant GenBank and EMBL databases by using the BLAST [23] network service at the National Center for Biotechnology Information and the European Biotechnology Information. ClustalW was used for multiple-sequence alignments [24].

### 3.4. Plasmid Constructions and Mutant Complementation Studies

For complementation studies, the *P. mirabilis* R110 gene *eptC* (PMI3104) was PCR amplified by using two specific oligonucleotides F1*eptC* (5′-TGGCTGGATATGAGCATTCA-3′) and R1*eptC* (5′-CCAGGTATGATGGCGGTAAG-3′) and chromosomal strain R110 DNA as template, ligated to plasmid pGEMT (Promega), and transformed into *E. coli* DH5α. Transformants were selected on LB plates containing ampicillin. Once checked, the plasmid with the amplified *eptC* gene (pGEMT-*eptC*) was transformed into *K. pneumoniae* core LPS mutants.

Recombinant plasmid pBAD18-Cm-*eptC* was obtained by pGEMT-*eptC* double digestion with *Sal*I and *Pvu*II and subcloning into pBAD18-Cm doubly digested with SalI and SmaI. This construct was transformed into *K. pneumoniae* core LPS mutants and *eptC* was expressed from the arabinose-inducible and glucose-repressible pBAD18-Cm promoter [20]. Repression from the *araC* promoter was achieved by growth in medium containing 0.2% (*w*/*v*) d-glucose, and induction was obtained by adding l-arabinose to a final concentration of 0.2% (*w*/*v*). The cultures were grown for 18 h at 37 °C in LB medium supplemented with chloramphenicol and 0.2% glucose, diluted 1:100 in fresh medium (without glucose), and grown until they reached an A_600_ of about 0.2. Then, l-arabinose was added, and the cultures were grown for another 8 h. Repressed controls were maintained in glucose-containing medium.

### 3.5. LPS Isolation and SDS-PAGE

For screening purposes, LPS was obtained after proteinase K digestion of whole cells [25]. LPS samples were separated by sodium dodecyl sulfate-polyacrylamide gel electrophoresis (SDS-PAGE) or Tricine (*N*-[2-hydroxy-1,1-bis(hydroxymethyl)ethyl]glycine)-SDS-PAGE and visualized by silver staining as previously described [25].

### 3.6. Large-Scale Isolation and Mild-Acid Degradation of LPS

Dried bacterial cells in 25 mM Tris–HCl buffer containing 2 mM CaCl_2_, pH 7.63 (10 mL·g^−1^), were treated at 37 °C with RNase and DNase (24 h, 1 mg·g^−1^ each), and then with proteinase K (36 h, 1 mg·g^−1^). The suspension was dialyzed and lyophilized, and the LPS was extracted with aqueous phenol [26]. After dialysis of combined water and phenol layers, contaminants were precipitated by adding 50% aqueous CCl_3_CO_2_H at 4 °C, the supernatant was dialyzed and lyophilized to give the LPS. A portion of the LPS was degraded with 2% acetic acid for 2 h at 100 °C, a precipitate was removed by centrifugation (13,000× *g*, 20 min), and the supernatant was fractionated on a column (50 × 2.5 cm) of Sephadex G-50 Superfine in 0.05 M pyridinium acetate buffer (4 mL pyridine and 10 mL acetic acid in 1 L of water) with monitoring using a differential refractometer (Knauer, Berlin, Germany).

### 3.7. Mass Spectrometry and NMR Studies

High-resolution electrospray ionization mass spectrometry was performed in the negative ion mode using a microTOF II instrument (Bruker Daltonics, Billerica, MA, USA). A sample of the OS (~50 ng·μL^−1^) was dissolved in a 1:1 (*v*/*v*) water–acetonitrile mixture and sprayed at a flow rate of 3 μL·min^−1^. Capillary entrance voltage was set to 4.5 kV and exit voltage to −150 V; drying gas temperature was 180 °C.

NMR spectra were obtained on a Bruker DRX-500 spectrometer (Bruker Nano GmbH, Berlin, Germany) using standard Bruker software at 30 °C in 99.95% D_2_O. Prior to the measurements, samples were deuterium-exchanged by freeze-drying twice from 99.9% D_2_O. A 150-ms duration of MLEV-17 spin-lock was used in two-dimensional TOCSY experiments. Chemical shifts are referenced to internal sodium 3-trimethylsilylpropanoate-2,2,3,3-d_4_ (δ_H_ 0) or acetone (δ_C_ 31.45).

## 4. Conclusions

A phylogenetic tree constructed from an alignment between PMI3040, PMI3576, PMI3104 (*eptC*), and representative known and putative PEtN transferases allowed us the identification of putative PEtN transferases in a proteomic search of the genome strain. The chemical data obtained in this work (Figures 5 and 6, and Table 1) clearly establish the role of *eptC* (PMI3104) product in the transfer of PEtN to the *O*-6 of l,d-Hep II in *P. mirabilis* strains. The *eptC* gene is found in all the *P. mirabilis* strains analyzed in this study. *eptC* homologues appear to be limited to species of the *Proteus* phylogenetic related genus *Photobacterium* and *Providencia.* The presence of a PEtN moiety linked to the *O*-6 position of l,d-Hep II has been shown in several *Providencia* strains.

The other two putative PEtN transferases PMI3576 and PMI3040 need further investigation. PMI3576 appears phylogenetically related to EptB and to a lesser degree to EptA of several *Enterobacteriaceae* species (Figure 2). EptA, also known as PmrC, transfers a PEtN moiety to the phosphate at the 1 and/or 4′ positions of lipid A [14]. No PEtN substituting lipid A was found in *P. mirabilis* grown in standard conditions. EptB transfers PEtN to the Kdo II residue of the inner core in *E. coli* [13], but no such Kdo II modification has been described in *P. mirabilis* core LPS [6,16]. The presence of a moiety of PEtN linked to Kdo II in minor *P. mirabilis* core molecules or when these strains are grown in non-standard conditions cannot be ruled out. The above considerations suggest that PMI3576 could be a PEtN transferase more likely involved in Kdo than in lipid A modification. At this time no function can be hypothesized for PMI3040.

## Figures and Tables

**Table 1.** ^1^H- and ^13^C-NMR chemical shifts (δ, ppm) of the modified core OS from *K. pneumoniae* 52145Δ*wabG* plus pBAD18-Cm-*eptC*.

Sugar residue	*C*-1; *H*-1	*C*-2; *H*-2	*C*-3; *H*-3 (*H*-3a,3e)	*C*-4; *H*-4	*C*-5; *H*-5	*C*-6; *H*-6 (*H*-6a,6b)	*C*-7; *H*-7 (*H*-7a,7b)	*C*-8; *H*-8 (*H*-8a,8b)
β-Glcp-(1→	103.7; 4.55	74.9; 3.32	76.7; 3.52	71.2; 3.37	77.5; 3.47	62.7; 3.97, 3.74		
α-ldHepp6P-(1→	102.5; 5.34	70.9; 4.15	71.5; 3.91	67.2; 3.91	72.4; 3.79	74.6; 4.57	62.5; 3.76, 3.85	
→3,4)-α-ldHepp-(1→	102.2; 5.08	71.4; 4.17	74.8; 4.15	74.9; 4.26	72.5; 4.15	69.5; 4.13	64.4; 3.72, 3.74	
→5)-α-Kdop	n.f.	97.0	35.4; 2.11, 1.94	67.0; 4.12	75.9; 4.12	72.3; 3.88	70.3; 3.70	64.4; 3.81, 3.65
NH_2_CH_2_CH_2_O-	63.3; 4.17	41.2; 3.31						

Assignment was performed using a two-dimensional ^1^H, ^13^C-HSQC experiment. n.f., not found. The ^31^P NMR signal is at δ 0.5.

**Table 2.** Bacterial strains and plasmids used.

Strain or plasmid	Relevant characteristics	Reference or source
*P. mirabilis*		
HI4320	Wild type	H.L.T. Mobley a
S1959	Wild type, serovar O3	Z. Sydorckzyk
R110	Rough mutant of strain S 1959	Z. Sydorckzyk b
51/57	Serovar O28	Z. Sydorckzyk b
50/57	Serovar O27	Z. Sydorckzyk b
14/57	Serovar O6	Z. Sydorckzyk b
TG83	Serovar O57	Z. Sydorckzyk b
*K. pneumoniae*		
52145Δ*wabH*	Non-polar *wabH* mutant	[9]
52145Δ*wabG*	Non-polar *wabG* mutant	[16]
*E. coli*		
DH5α	F^−^ *endA hsdR17* (r_k_^−^ m_k_^−^) *supE44 thi-1 recA1 gyr-A96 φ*80*lacZ*	[19]
Plasmid		
pGEMT easy	PCR generated DNA fragment cloning vector Amp^R^	Promega c
pGEMT-*eptC*	pGEMT with *eptC* from strain HI4320, Ap^R^	This study
pBAD18-Cm	Arabinose-inducible expression vector, Cm^R^	[20]
pBAD18-Cm-*eptC*	Arabinose-inducible *eptC*, Cm^R^	This study

Locations:

a University of Michigan, MI, USA;

b National Microbiology Institute, Warsaw, Poland;

c Barcelona, Spain.

## Abbreviations

LPS: lipopolysaccharide
OS: oligosaccharide
*O*-PS: *O*-polysaccharide or *O*-antigen
PEtN: phosphoethanolamine
l,d-Hep: l*-glycero-*d*-manno-*heptose
Kdo: 3-deoxy-d-manno-oct-2-ulosonic acid or 3-deoxyoctulosonic acid
l-Ara4N: 4-amino-4-deoxy-l-arabinose
